# Supplementary figures and images for: STENCIL: A web templating engine for visualizing and sharing life science datasets
Source: PLoS Comput Biol. 2022 Feb 9;18(2):e1009859. doi: 10.1371/journal.pcbi.1009859 (PMC8863220; doi:10.1371/journal.pcbi.1009859)

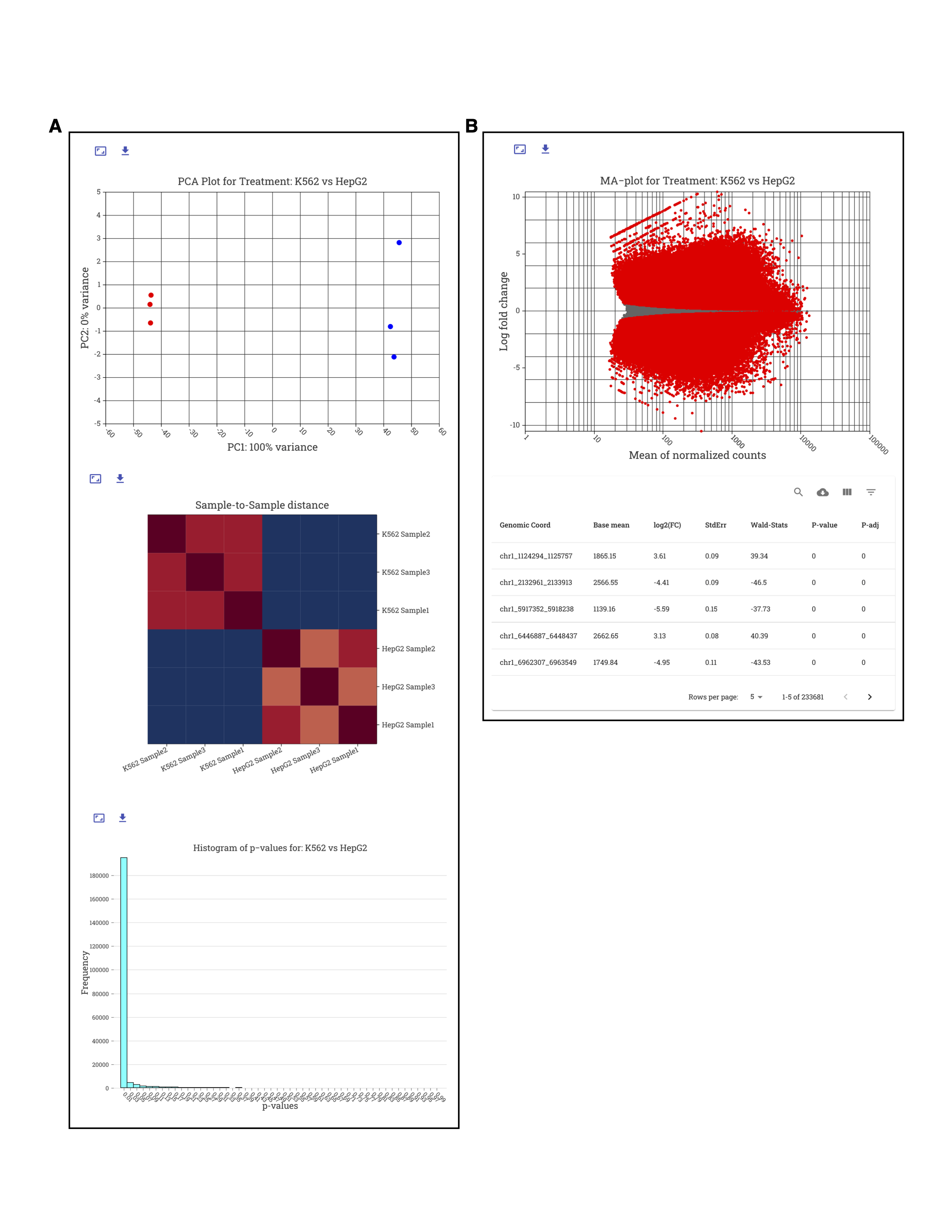

Supplement: S1 Fig — (A) DESeq2 charts are visualized as dynamic charts in STENCIL. All data is generated and hosted directly from Galaxy. (B) The nivo charting library allows for on-the-fly generated of interactive plots containing hundreds of thousands of unique datapoint in seconds. (TIFF) [file pcbi.1009859.s001.tiff]
